# A serum microRNA signature as a prognostic factor for patients with advanced NSCLC and its association with tissue microRNA expression profiles

**DOI:** 10.3892/mmr.2021.12046

**Published:** 2021-03-29

**Authors:** Jing Guo, Rui Meng, Zhongyuan Yin, Pengcheng Li, Rui Zhou, Sheng Zhang, Xiaorong Dong, Li Liu, Gang Wu

Mol Med Rep 13: 4643-4653, 2016; DOI: 10.3892/mmr.2016.5114

Subsequently to the publication of this paper, an interested reader drew to the authors' attention that, in Fig. 5B on p. 4651, the images selected to represent the miR-486/mimic and miR-221/inhibitor conditions for the migration experiments with the H358 cell line bore some striking similarities. After having examined their original data, the authors realized that they uploaded an incorrect image to represent the miR-486/mimic data panel during the process of compiling this figure.

The corrected version of Fig. 5, showing all the correct data for Fig. 5B, is shown on the next page. Note that the replacement of the erroneous data does not affect either the results or the conclusions reported in this paper, and all the authors agree to this Corrigendum. The authors are grateful to the Editor of *Molecular Medicine Reports* for granting them this opportunity to publish a Corrigendum, and apologize to the readership for any inconvenience caused.

## Figures and Tables

**Figure 5.**
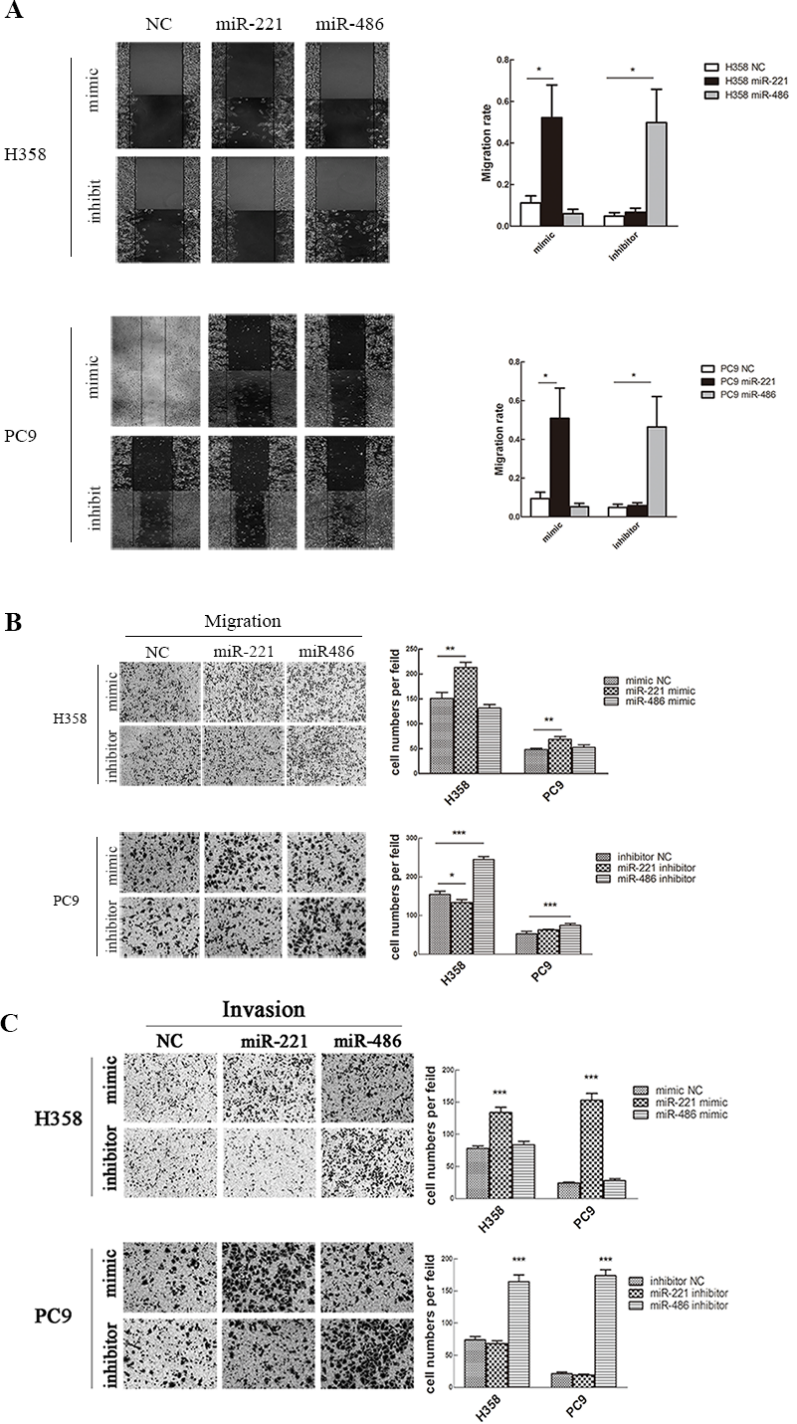
Upregulation of miR-221 or downregulation of miR-486 promotes cell migration and invasion. (A) Wound healing assay and (B) Transwell migration assay were used to measure cell migratory capacity. (C) Transwell invasion assay was used to measure cell invasive capacity. Magnification, ×200. Data are presented as the mean ± standard deviation. *P<0.05, **P<0.01, ***P<0.001. miR, microRNA; NC, negative control.

